# Supplementary material for: Computational modelling of hip resurfacing arthroplasty investigating the effect of femoral version on hip biomechanics
Source: PLoS One. 2021 May 27;16(5):e0252435. doi: 10.1371/journal.pone.0252435 (PMC8158908; doi:10.1371/journal.pone.0252435)
Supplement: Attachment — Submitted filename: Response to Reviewers_PLOSone.pdf [file pone.0252435.s001.pdf]

January 23, 2021

Jose Manuel Garcia Aznar
Academic Editor
PLOS ONE

Dear Dr. Anzar,

RE: PONE-D-20-34959.

Thank you for reviewing the manuscript of our original research article entitled "Computational Modelling of Hip Resurfacing Arthroplasty Investigating the Effect of Femoral Version on Hip Biomechanics" for consideration for publication in the PLOS One.

After careful review of PLOS ONE's publication criteria, we have edited our manuscript according to the journal's standards. We hope you find the corrections within our revised manuscript acceptable. Notable changes include:

- Level 1 heading used for all major sections. Bold type, 18pt font.
- Level 2 heading used for all sub-sections of major sections. Bold type, 16pt font.
- Figure citation corrected across all the text.
- Each figure caption now appears directly after the paragraph in which they are first cited.
- All figure titles are now bolded.
- Tables are now presented directly after the paragraph in which they are first cited.
- Tables are all cell-based.
- References with more than six authors now list the first six author names, followed by "et al."

The authors would also like to thank the reviewer and academic editor for their comments. We value your insightful input and have attempted to edit our manuscript in a way that reflects these.

Reviewer 1 comment: The introduction presents a lack of other previous published works related with hip resurfacing offset and position.

Author response: There is unfortunately a lack of scientific evidence regarding the impact of femoral retroversion on hip biomechanics and the impact of this hip resurfacing. The purpose of our Finite Element Analysis was to explore this in a computational model given the paucity of research in this field. Insight on femoral retroversion on HRA with respect to impingement and force transmission across the hip joint may encourage further clinical research in this field.

In light of your comment, we have edited our introduction in order to highlight this issue more clearly. We hope you appreciate the new changes.

Reviewer 1 comment: The materials and methods presents a lack of information to understand the simulation and the results. Some important technical aspects are critical to analyze the results.

Author response: We have reviewed our Materials and Methods section of our paper. The authors believed to have successfully explained both the computational model techniques used to create the finite element analysis as well as the different models analysed. Did the reviewer feel strongly about any specific aspect of the methods that may need clarity?

However, we have edited some parts of the methods in order to improve the understanding of our approach. We believe this now reads better and may facilitate understanding. Most of these edits are found in the last two paragraphs of the methods.

The authors wishes to thank both the reviewer and academic editor for their time in reviewing these changes. We hope these are satisfactory to proceed with publication in PLOS One. Should you have further comments of concerns, please feel free to contact us.

There have been no changes to our financial disclosures.

Thank you for your consideration.

Sincerely,

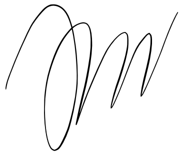

Jonathan Bourget-Murray, MD CM FRCSC
Senior Orthopaedic Trauma Fellow
John Radcliffe Hospital | Oxford University Hospitals NHS Trust
University of Oxford, Oxford, UK
